# Optical Coherence Tomography-Guided vs. Angiography-Guided Percutaneous Coronary Intervention for Complex Coronary Lesions: A Systematic Review and Meta-Analysis

**DOI:** 10.3390/diagnostics15151907

**Published:** 2025-07-30

**Authors:** Muhammad Hamza Shuja, Muhammad Ahmed, Ramish Hannat, Laiba Khurram, Hamza Ali Hasnain Sheikh, Syed Hasan Shuja, Adarsh Raja, Jawad Ahmed, Kriti Soni, Shariq Ahmad Wani, Aman Goyal, Bala Pushparaji, Ali Hasan, Raheel Ahmed, Hritvik Jain

**Affiliations:** 1 Department of Internal Medicine, Dow University of Health Sciences, Karachi 74200, Pakistan; hamzashuja9825@gmail.com (M.H.S.); hasanshuja6@gmail.com (S.H.S.); 2 Department of Internal Medicine, Shaheed Mohtarma Benazir Bhutto Medical College Lyari, Karachi 75660, Pakistan; muhammadahmed5515@gmail.com (M.A.); laibakhurram.doctor@gmail.com (L.K.); hamzaalishaekh10@gmail.com (H.A.H.S.); adarshbudhwani01@gmail.com (A.R.); 3 Department of Internal Medicine, Services Institute of Medical Sciences, Lahore 54000, Pakistan; ramish.hannat@gmail.com; 4 Department of Internal Medicine, Northwest Health—Porter, Valparaiso, IN 46383, USA; jawadahmedd13@gmail.com; 5 Department of Internal Medicine, SUNY Upstate Medical University, Syracuse, NY 13210, USA; kritisoni93@gmail.com; 6 Department of Internal Medicine, Detroit Medical Center, Wayne State University, Detroit, MI 48202, USA; shariqqwani12@gmail.com; 7 Department of Internal Medicine, Cleveland Clinic Foundation, Cleveland, OH 44195, USA; goyala7@ccf.org; 8 Department of Cardiology, MetroHealth Medical Center, Cleveland, OH 44109, USA; bpushparaji@metrohealth.org; 9 Department of Cardiology, National Heart and Lung Institute, Imperial College London, London SW7 2AZ, UK; ali.hasan21@imperial.ac.uk; 10 Department of Cardiology, All India Institute of Medical Sciences, Jodhpur 342005, India; hritvikjain2001@gmail.com

**Keywords:** percutaneous coronary intervention, optical coherence tomography, angiography, major adverse cardiac events, meta-analysis

## Abstract

**Background**: Despite advances in coronary artery disease (CAD) treatment, challenges persist, particularly in complex lesions. While percutaneous coronary intervention (PCI) is widely used, its outcomes can be affected by complications like restenosis. Optical coherence tomography (OCT), offering higher-resolution imaging than angiography, shows promise in guiding PCI. However, meta-analytical comparisons between OCT-guided and angiography-guided PCI remain limited. **Methods**: Databases, including PubMed, Scopus, Cochrane Library, and ClinicalTrials.gov, were queried through May 2025 to identify randomized controlled trials (RCTs) comparing OCT-guided PCI with angiography-guided PCI. Data were pooled using risk ratios (RRs) and mean difference (MD) with 95% confidence intervals (CIs) in a random-effects model. **Results**: Five RCTs involving 5737 patients (OCT: 2738 and angiography: 2999) were included. On pooled analysis, OCT-guided PCI was associated with a notable reduction in major adverse cardiovascular event (MACE) (RR: 0.71, *p* = 0.0001), cardiac mortality (RR: 0.43, *p* = 0.003), target lesion revascularization (TLR) (RR: 0.53, *p* = 0.007), and stroke (RR: 0.17, *p* = 0.02), compared to angiography-guided PCI. No significant differences were noted for all-cause mortality and myocardial infarction. **Conclusions**: In patients with complex coronary lesions, OCT-guided PCI reduces the risk of MACE, cardiac mortality, TLR, and stroke, compared to angiography-guided PCI only. This study supports incorporating advanced imaging techniques like OCT to improve clinical outcomes, especially in complex PCIs.

## 1. Introduction

Coronary artery disease (CAD) is the third most prevalent cause of mortality, with over 17.8 million deaths every year [1]. CAD can manifest as myocardial infarction (MI), stable and unstable angina, and silent ischemia. Complex CAD lesions, such as bifurcation plaques, highly calcified segments, and chronic total occlusions (CTOs), present significant procedural challenges [2]. Percutaneous coronary intervention (PCI) continues to be one of the most widely used methods for the management of CAD. Nevertheless, PCI has certain limitations, including risks of restenosis, stent thrombosis, and procedural complications in complex cases [3]. Angiography, a long-standing technique for diagnosing and treating CAD, involves injecting contrast dye into blood vessels and using X-rays to visualize coronary anatomy [4].

Angiography before PCI is effective in providing information on the intraluminal vessel diameter, length, and diameter of the coronary artery lesions, and helps in deciding the dimensions of the coronary artery stents. Angiography is also effective if used after PCI for visualizing the stent restenosis or for deciding the post-dilatation balloon diameter and inflation pressure [4]. However, the angiography technique has certain limitations.

Several ambiguities may affect the clarity of angiographic images [5]. One of these challenges is the overlap of components, where the complex anatomy makes it challenging to distinguish the main vessel from the smaller side branches. The actual lengths and angles of the regions affected may be changed due to foreshortening and result in a wrong interpretation. Additionally, vessel tortuosity complicates accurate assessment due to the curved and twisted anatomy of the arteries. Furthermore, incomplete opacification by the contrast agent may obscure the lesion or adjacent vessels, resulting in diagnostic uncertainty [6,7]. The size and shape of the arteries, as well as prominent calcification near the bifurcation, may also make angiogram interpretation more difficult, or impair quality [6,7]. These uncertainties may significantly affect critical aspects of stent placement. Angiography gives only a two-dimensional, intraluminal image of the coronary arteries, does not provide information on characteristics of the coronary artery plaque, coronary artery dissection after PCI, or the extent of the involvement of the vessel wall in a coronary artery lesion.

Optical coherence tomography (OCT) is an intravascular imaging technique that uses light rays to form a cross-sectional, high-resolution image of the coronary arteries and provides the clinician with both intraluminal and vascular wall characteristics of coronary arteries and their associated lesions. OCT can be used to overcome these shortcomings of angiography. Because of the ability of the OCT to give both intraluminal and vascular wall characteristics, it can more accurately predict the plaque morphology, calcifications in vessel walls, dimensions of lesions, and stent dimensions. OCT may have a few drawbacks, like less clinical understanding due to the technique being new, high operator skills, a small field of view, and decreased patient cooperation [4,8].

OCT has been regarded as superior to angiography for guiding PCI; however, it is unclear how OCT compares to angiography-guided methods when managing complex coronary lesions. Therefore, this systematic review and meta-analysis aims to compare clinical outcomes between OCT-guided PCI and angiography-guided PCI in patients with complex coronary lesions.

## 2. Materials and Methods

The systematic review and meta-analysis were conducted and reported per the updated Preferred Reporting Items for Systematic Reviews and Meta-Analyses (PRISMA) 2020 guidelines [9] (Table S1). The search strategy and protocol of this study were registered prospectively on the International PROSPERO Registry for Systematic Review (Identification No.: CRD42024599058).

### 2.1. Data Sources and Search Strategy

A literature search of Pubmed, Scopus the Cochrane Library, and ClinicalTrials.gov was conducted from inception through May 2025. Broad and specific MeSH terms such as “percutaneous coronary intervention”, “angiography”, “coronary lesions”, “complex coronary lesion”, “optical coherence tomography”, and “OCT” were utilized to create search strings. Boolean operators like “AND”, “OR”, and “NOT” were used to refine the search and ensure thorough coverage (Table S2).

### 2.2. Study Selection

After importing all relevant articles into Mendeley Reference Manager and removing duplicates, two investigators (M.H.S. & R.H.) independently reviewed the titles and abstracts, followed by full-text reviews. The inclusion criteria included the following: (i) randomized controlled trials (RCTs) involving adults (>18 years), (ii) undergoing PCI for complex coronary lesions, (iii) OCT-guided PCI in one arm, (iv) angiography-guided PCI in the other arm, and (v) reported one of the outcomes of interest. Studies that were not RCTs, not comparing OCT-guided PCI to angiography-guided PCI, and not reporting relevant outcomes were excluded. Discrepancies were resolved by consulting an independent third reviewer (L.K.).

### 2.3. Data Extraction

Using a pre-piloted Microsoft Excel spreadsheet, the following data were extracted: author name, publication year, clinical trial number, country, total patient population, OCT-guided patient population, angiography-guided patient population, mean age, percentage of female/male patients, body mass index (BMI), hypertension, diabetes mellitus, smoking status, left ventricular ejection fraction (LVEF), and follow-up duration (years).

### 2.4. Outcomes of Interest

The primary endpoints of this meta-analysis were major adverse cardiovascular events (MACEs) and cardiac mortality. Secondary endpoints included target lesion revascularization (TLR), stroke, stent thrombosis, all-cause mortality, procedural duration, ischemia-driven target-vessel revascularization (TVR), minimal stent area (MSA), and myocardial infarction (MI). The definition of MACE in each study and type of complex lesions is depicted in Table 1.

### 2.5. Assessment of Study Quality

The risk of bias in the RCTs was independently assessed by two investigators (H.A.H.S. & R.H.) using the Cochrane Risk of Bias 2 (RoB 2) tool [10]. The evaluation included the following domains: (1) random sequence generation, (2) allocation concealment, (3) blinding of participants and personnel, (4) blinding of outcome assessment, (5) incomplete outcome data, (6) selective outcome reporting, and (7) other potential sources of bias. Each RCT was thoroughly reviewed and classified as having a “low risk”, “unclear risk”, or “high risk” of bias.

### 2.6. Statistical Analysis

The results were presented as risk ratios (RRs) or mean differences (MD) with 95% confidence intervals (CIs) and were pooled using a DerSimonian–Laird random effects model. Forest plots were generated to visually depict the results. A *p*-value less than 0.05 was considered statistically significant. The Higgins’ I2 statistic was used to quantify heterogeneity between studies. Low heterogeneity was defined as an I2 value < 50%, moderate heterogeneity as a value between 50% and 75%, and high heterogeneity as a value > 75%. We also performed a sensitivity analysis using the leave-one-out method for outcomes demonstrating high heterogeneity [11]. All statistical analyses were performed using Review Manager (version 5.3; Copenhagen: The Nordic Cochrane Centre, The Cochrane Collaboration, 2014).

**Table 1:** Table of characteristics of included trials.

Trial Name	Country	Definition of MACE	Definition of Complex Lesion	Primary Outcome/Major Outcomes
ILLUMEN IV [12]	Europe, North America, Asia, Oceania	The Composite of Cardiac Death, Target-Vessel MI, or Definite/Probable Stent Thrombosis	Long or Multiple Lesions with intended Total Stent Length more than or equal to 28 mm, Bifurcation Lesion with Stenting Intended in both the Main and Side Branches more than or equal to 2.5 mm in Diameter, Severe Target Lesion Calcification, defined as Angiographically Visible Calcification on both sides of the Vessel Wall in the absence of Cardiac Motion, Chronic Total Occlusion (CTO) or Diffuse or Multifocal In-Stent Restenosis.	Final Post-PCI Minimal Stent Area (MSA) assessed by OCT in both groups, The 2-Year Outcome of MACE, The 2-Year Effectiveness Outcome of Target-Vessel Failure (TVF).
OCTOBER [13]	Europe	Composite of Death from a Cardiac Cause, Target Lesion Myocardial Infarction, or ischemia-Driven Target Lesion Revascularization at a median follow-up of 2 years.	Complex Coronary Artery Bifurcation Lesion.	MACE
OCCUPI [14]	South Korea	Composite of Cardiac Death, Myocardial Infarction, Stent Thrombosis, or Ischaemia-Driven Target-Vessel Revascularization 1 Year after PCI.	Acute Myocardial Infarction, Chronic Total Occlusion, Long Lesion [Expected Stent Length ≥28 mm based on Angiography], Calcified Lesion, Bifurcation Lesion, Unprotected Left Main Disease, Small Vessel Diseases [Vessel Diameter <2.5 mm], Intracoronary Thrombus visible on Angiography, Stent Thrombosis, In-Stent Restenosis or Bypass Graft Lesion.	MACE
CALIPSO [15]	France	Composite of Cardiovascular death, any Myocardial Infarction or need for Clinically Driven Reintervention on the Target Lesion.	Stable Moderate-to-Severe Calcified Coronary Lesions on Coronary Angiography Scheduled for PCI.	Minimal Stent Area (MSA) on the Qualifying OCT run.
RENOVATE- COMPLEX- PCI [16,17]	South Korea	-	Complex Coronary Artery Lesions were defined as true Bifurcation Lesions according to the Medina classification system12 with a Side-Branch Diameter of at least 2.5 mm; a Chronic Total Occlusion; Unprotected Left Main Coronary Artery Disease; Long Coronary Artery Lesions that would involve an expected Stent Length of at least 38 mm; Multi Vessel PCI involving at least two Major Epicardial Coronary Arteries being treated at the same time; a lesion that would necessitate the use of multiple stents (at least three planned stents); a lesion involving In-Stent Restenosis; a Severely Calcified Lesion; or Ostial Lesions of a Major Epicardial Coronary Artery.	The primary end point was Target-Vessel Failure, which was defined as a composite of death from Cardiac Causes, Target-Vessel–Related Myocardial Infarction, or Clinically Driven Target-Vessel Revascularization

## 3. Results

### 3.1. Study Characteristics and Baseline Demographics

**Figure 1:**
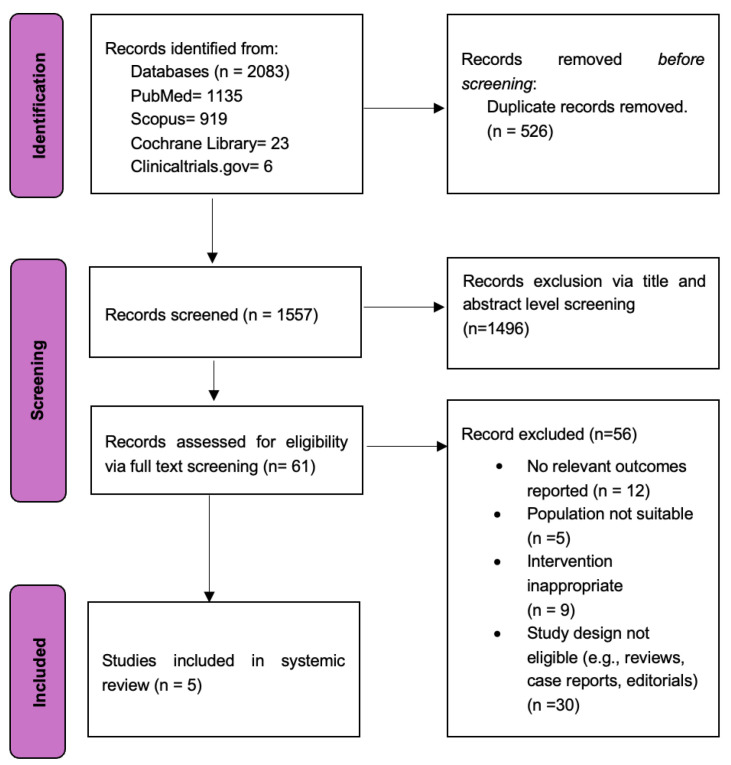
PRISMA 2020 flowchart.

The initial systematic literature search identified 2083 studies, from which five RCTs [12,13,14,15,16,17] were selected for inclusion in this meta-analysis (Figure 1). Outcome data were extracted and pooled from a total of 5737 patients (2738: OCT-guided PCI and 2999: angiography-guided PCI). Table 1 and Table 2 provide more information on the study and its baseline characteristics.

### 3.2. Quality Assessment

**Figure 2:**
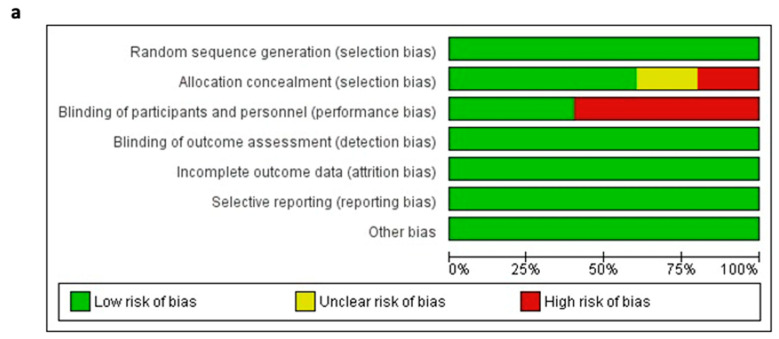

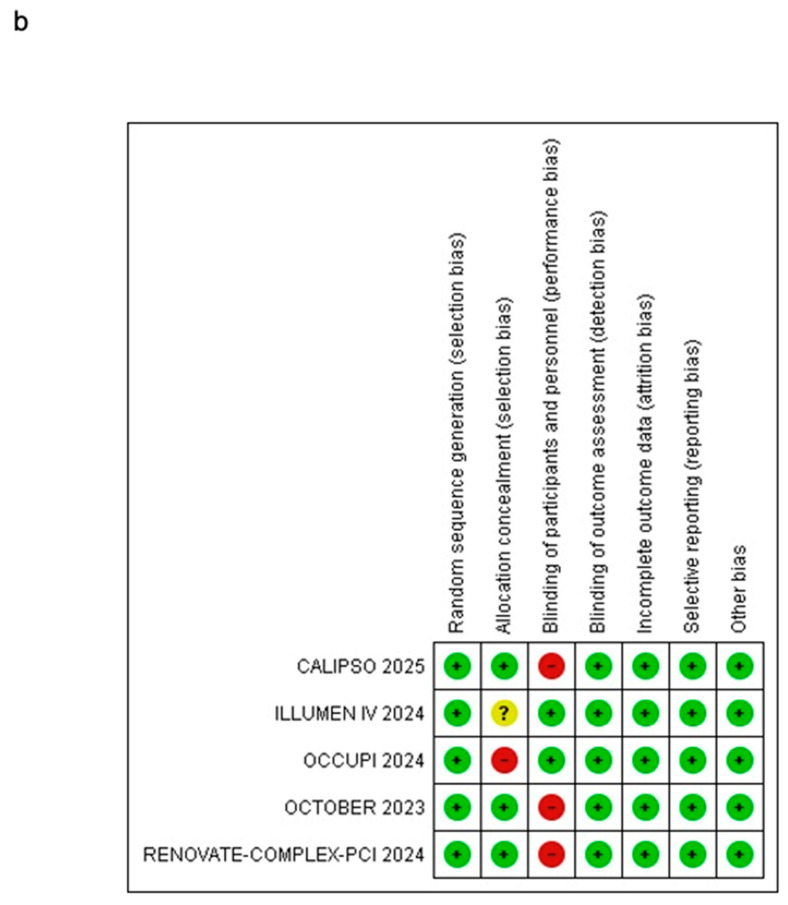
Risk of bias analysis for the included randomized controlled trials. (**a**) Risk of bias graph. (**b**) Risk of bias summary. Symbols represent the judgment for each domain: “+” means low risk of bias; “−” means high risk of bias; “?” means unclear risk of bias.

Across the five RCTs, most domains showed a low risk of bias. Random sequence generation, allocation concealment, outcome assessment, handling of incomplete data, selective reporting, and other biases were consistently well-managed using sound methodologies. However, the blinding of participants and personnel was a notable limitation. Four of the five studies were open-label, leading to a high risk of performance bias. Only ILLUMEN IV employed a single-blinded design, minimizing this concern (Figure 2a,b and Table S3).

### 3.3. Primary Endpoints

**Figure 3:**
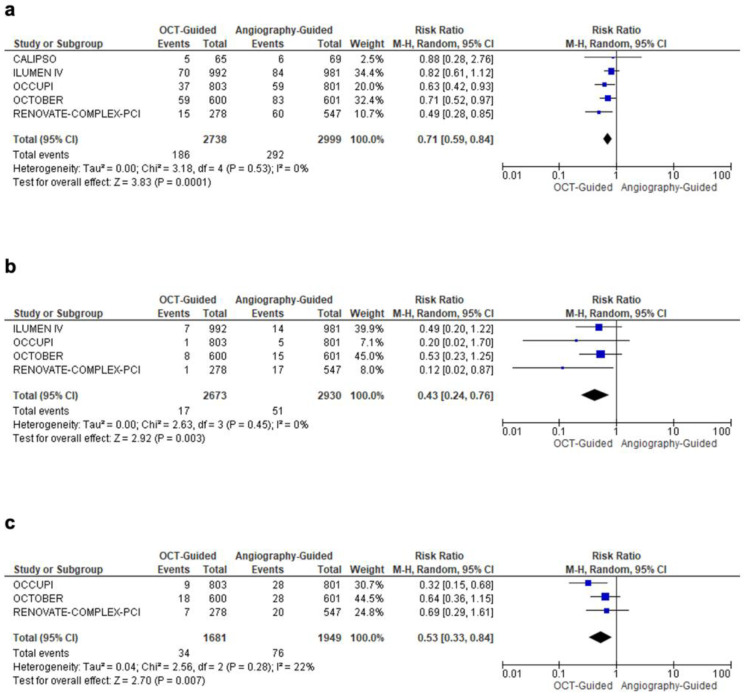
Forest plot comparing optical coherence tomography versus angiography-guided percutaneous coronary intervention for complex coronary lesions. (**a**) Major adverse cardiovascular events. (**b**) Cardiovascular mortality. (**c**) Target lesion revascularization.

On pooled analysis from all RCTs, OCT-guided PCI was associated with a significantly lower risk of MACE by 29% (RR: 0.71; 95% CI: 0.59, 0.84; I^2^ = 0%; *p* = 0.0001) (Figure 3a). Similarly, cardiac mortality was also significantly lower by 57% in patients undergoing OCT-guided PCI (RR: 0.43, 95% CI: 0.24, 0.76; I^2^ = 0%; *p* = 0.003) compared to angiography-guided PCI (Figure 3b).

### 3.4. Secondary Endpoints

**Figure 4:**
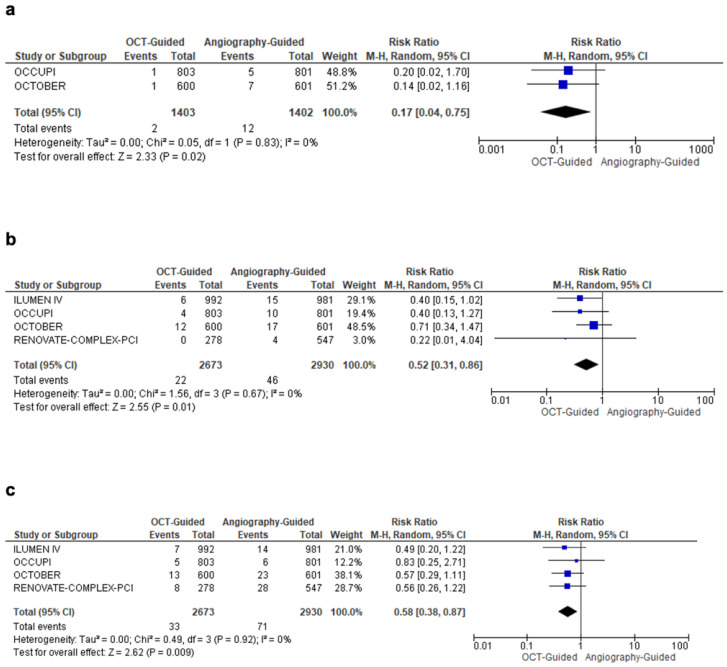
Forest plot comparing optical coherence tomography versus angiography-guided percutaneous coronary intervention for complex coronary lesions. (**a**) Stroke. (**b**) Stent thrombosis. (**c**) All-cause mortality.

OCT-guided PCI was associated with a 47% reduction in total target lesion revascularization (TLR) (RR = 0.53; 95% CI: 0.33, 0.84; I^2^ = 22%; *p* = 0.007) across three studies (Figure 3c). In addition, OCT guidance led to an 83% reduction in the risk of stroke (RR = 0.17; 95% CI: 0.04, 0.75; I^2^ = 0%; *p* = 0.02), a 48% reduction in stent thrombosis (RR = 0.52; 95% CI: 0.31, 0.86; I^2^ = 0%; *p* = 0.01), and a 42% reduction in all-cause mortality (RR = 0.58; 95% CI: 0.38, 0.87; I^2^ = 0%; *p* = 0.009) (Figure 4a–c). OCT-guided PCI was associated with a significantly longer total procedural duration by around 16 minutes (MD: 16.14; 95% CI: 6.67, 25.61; I^2^ = 96%; *p* = 0.0008) compared to angiography-guided PCI. Due to high heterogeneity, a sensitivity analysis was performed after excluding the OCTOBER trial (MD: 11.67; 95% CI: 4.99, 18.36; I^2^ = 89%; *p* = 0.0006). However, no significant differences in ischemia-driven TVR were observed (RR: 0.67; 95% CI: 0.44, 1.03; I^2^ = 55%; *p* = 0.07). A further sensitivity analysis for ischemia-driven TVR, excluding the OCCUPI trial, also showed no significant difference (RR: 0.84; 95% CI: 0.63, 1.12; I^2^ = 0%; *p* = 0.23). Similarly, MSA (MD: 0.93; 95% CI: −0.37, 2.23; I^2^ = 94%; *p* = 0.16) and MI (RR: 0.77; 95% CI: 0.57, 1.03; I^2^ = 29%; *p* = 0.07) did not differ significantly between the groups (Figures S1–S4). A complete overview of all primary and secondary outcomes is summarized in Table 3.

## 4. Discussion

This meta-analysis of five RCTs with 5737 patients reports that OCT-guided PCI had significantly lower risks of MACE, cardiac mortality, all-cause mortality, stroke, and stent thrombosis compared to angiography-guided PCI [12,13,14,15,16,17]. Revascularization outcomes, such as TLR, were also improved with OCT, while ischemia-driven TVR and MI showed no significant differences. However, OCT-guided PCI had a significantly longer total procedural duration compared to angiography-guided PCI.

Intravascular imaging offers superior cross-sectional visualization of coronary arteries compared to angiography, leading to improved long-term clinical outcomes, particularly in complex cases. Complex coronary artery lesions, including chronic total occlusions, bifurcation lesions [18,19,20], and heavily calcified lesions [21], present unique challenges during PCI due to their intricate anatomy and the associated higher risks for adverse outcomes such as MI and repeat revascularization. Relying solely on angiographic imaging for these lesions can result in insufficient evaluation of critical factors that influence procedural success. Trials such as IVUS-XPL (Impact of Intravascular Ultrasound Guidance on Outcomes of Xience Prime Stents in Long Lesions) and ULTIMATE (Intravascular Ultrasound-Guided Drug-Eluting Stents Implantation in “All-Comers” Coronary Lesions) have demonstrated significant benefits of IVUS in drug-eluting stent implantation, including reductions in cardiac events and mortality [22,23,24]. OCT is an advanced technique that provides high-resolution cross-sectional images that yield detailed insights into plaque morphology and stent deployment. By allowing for real-time assessment of stent apposition and expansion, OCT enhances the precision of interventions, which can lead to improved clinical outcomes [25]. Given the ability of OCT to identify lesions, malposition of stents, defects in the coronary vessel walls, and characteristics of the coronary plaques better than angiography, it has been identified that OCT has better predictive value in predicting unfavorable outcomes like MACE, stent thrombosis, all-cause mortality, and cardiovascular mortality [13]. Contrast-Associated Acute Kidney Injury (CA-AKI) in cardiovascular angiography has been a common complication, especially in patients with advanced chronic kidney disease (CKD) [26]. An article published in 2020 showed that if low molecular weight dextran (LMWD) is used, OCT-guided PCI can be performed in advanced CKD patients using minimal contrast [27]. However, studies have also reported that the OCT technique requires greater contrast volume as compared to the angiography technique, which is concerning for patients with decreased renal function [28].

Previous meta-analyses comparing OCT guidance to angiography alone have produced inconsistent results, largely due to methodological flaws. These include the inappropriate pooling of heterogeneous studies, particularly the inclusion of trials evaluating different imaging modalities (e.g., IVUS and OCT), or studies not primarily designed to assess PCI guidance strategies. For instance, Sreenivasan et al. [29] and Khan et al. [30] combined RCTs involving both IVUS and OCT, despite their distinct mechanisms and clinical implications. Similarly, Attar et al. [31] included the EROSION III (Optical Coherence Tomography-Guided Reperfusion in ST-Segment Elevation Myocardial Infarction with Early Infarct Artery Patency) trial, which focused on stent implantation in STEMI rather than the comparative efficacy of imaging guidance, potentially biasing results in favor of OCT. These methodological flaws reduce the reliability and specificity of the findings. These methodological shortcomings have hindered the ability to draw clear conclusions about the benefits of OCT. A recent meta-analysis by Ahmed et al. [32], which focused solely on OCT-guided PCI, reported reductions in cardiovascular mortality and stent thrombosis but found no notable differences in all-cause mortality, MACE, MI, TLR, or TVR compared to angiography. In contrast, our study specifically targets patients with complex lesions, providing the first comprehensive analysis of OCT in this cohort.

This current study underscores that OCT does not lead to significant reductions in MI, aligning with the findings of Ahmed et al. [32]. However, our analysis reveals noteworthy differences in patients with complex lesions. While Ahmed et al. found no significant effects on incidences of MACE, stent thrombosis, TLR, or stroke, our meta-analysis highlights a significant reduction in these outcomes as well as all-cause mortality for this high-risk patient subgroup. This contrast emphasizes the potential of OCT to improve outcomes in a patient population where complex lesions often present greater procedural challenges and higher risks for adverse events. The reduced risk observed in the OCT group can be attributed to several key advantages. OCT offers superior imaging resolution compared to traditional techniques like angiography, enabling more precise assessments of plaque morphology, stent placement, and vessel wall characteristics. Our analysis also reveals a reduction in cardiac mortality and stent thrombosis, with the latter being one of the most severe complications specific to PCI [33]. This finding is significant, as demonstrated in the ILUMIEN IV: OPTIMAL PCI (Optical Coherence Tomography-Guided Coronary Stent Implantation Compared with Angiography: A Multicenter Randomized Trial in PCI) trial, where 95.7% of patients who experienced stent thrombosis also suffered a myocardial infarction and subsequently died [8]. Thus, the reduced risk of stent thrombosis following OCT is crucial, offering valuable insights for clinicians in choosing between the two techniques.

The incorporation of OCT in PCI offers significant advantages in clinical scenarios to manage complex coronary lesions. With high-resolution imaging, OCT can detect subtle abnormalities such as thin cap fibroatheromas and incomplete stent apposition, which might be missed by conventional angiography [34]. This enhanced visualization allows for real-time adjustments during procedures, effectively reducing the risk of adverse outcomes like restenosis and stent thrombosis. Moreover, OCT’s precise vessel dimension measurements ensure optimal stent sizing and deployment, minimizing procedural complications and improving long-term outcomes. Additionally, the observed 83% reduction in stroke risk with OCT guidance highlights its role in reducing vascular injury and embolization during stent placement. Although no significant differences in MI were found, the observed safety and procedural efficacy improvements suggest that OCT guidance provides meaningful clinical benefits, especially for high-risk patients. However, these benefits come at the cost of increased total procedural duration, which on average was 16 min longer than angiography-guided PCI, likely due to the additional imaging steps, pullbacks, and interpretation time required for precise stent placement. While this may pose logistical challenges in high-volume centers, the trade-off may be justified by improved safety and efficacy in high-risk patients. These findings support the routine incorporation of OCT in complex PCI procedures. To maximize its potential, clinicians should be trained in OCT techniques, and healthcare systems should evaluate the cost-effectiveness of its widespread use. Future studies with extended follow-up and standardized protocols will be crucial in refining OCT’s role in improving patient outcomes and optimizing coronary artery disease management.

### Limitations

This meta-analysis has several important limitations. First, substantial heterogeneity was noted for some outcomes, particularly procedural duration (I^2^ = 96%) and ischemia-driven TVR (I^2^ = 55%). Sensitivity analyses excluding the OCTOBER and OCCUPI trial reduced this heterogeneity and yielded more stable estimates, but residual variability remained. Second, definitions of MACE and complex lesions differed markedly across trials. For example, OCCUPI used broader criteria, including thrombus and small-vessel disease, whereas OCTOBER focused solely on bifurcation lesions. These inconsistencies limited cross-trial comparability and contributed to clinical heterogeneity, further compounded by the small number of RCTs, which constrains generalizability. Third, individual patient data were unavailable, precluding subgroup analyses to explore differences in baseline comorbidities or patient-level modifiers of outcomes. Fourth, this review focused exclusively on OCT-guided PCI and did not include intravascular ultrasound (IVUS). Therefore, findings should not be generalized to all intravascular imaging techniques for complex coronary lesions. Fifth, OCT is a relatively newer modality compared with angiography, and clinical familiarity remains limited. Angiography benefits from decades of use, providing an experience advantage in both interpretation and procedural decision-making. OCT interpretation, on the other hand, is inherently more subjective and highly operator-dependent, emphasizing the importance of expertise for optimal results. Sixth, the characteristics of enrolled populations varied across trials, particularly regarding comorbidities (e.g., hypertension, diabetes, BMI) and the definition of “complex” lesions. For instance, OCCUPI classified multiple lesion types (e.g., thrombus, chronic total occlusion and bifurcation) as complex; CALIPSO restricted inclusion to calcified lesions; and ILLUMEN IV considered long or multiple lesions (≥28 mm stent length), bifurcations, severe calcification, CTOs, and diffuse in-stent restenosis. These differing inclusion criteria added another layer of heterogeneity. Seventh, crossover between treatment arms occurred in OCCUPI: 12 patients allocated to OCT-guided PCI underwent angiography-guided PCI, and 8 patients crossed over in the opposite direction. No crossover was reported in other trials. Finally, performance bias was an unavoidable concern: four of the five trials (OCTOBER, OCCUPI, CALIPSO, and RENOVATE COMPLEX PCI) were open-label, meaning participants and operators were aware of treatment allocation. This awareness may have influenced procedural diligence, co-interventions, and follow-up intensity, potentially affecting outcomes beyond the interventions themselves.

## 5. Conclusions

This analysis concludes that OCT-guided PCI leads to a significant reduction in MACE, TLR, stroke, and stent thrombosis compared to angiography-guided PCI in patients with complex lesions. The findings highlight the safety and efficacy of OCT in managing complex coronary lesions. Moving forward, it is essential to conduct larger, multicenter trials with extended follow-up periods to further assess the long-term benefits of OCT guidance, including its impact on mortality and repeat MI. Additionally, standardizing procedural protocols across studies will help address the observed heterogeneity and enhance the applicability of OCT in diverse patient populations. Continued research will be crucial in optimizing PCI strategies and improving patient outcomes in the context of complex lesions.

## Figures and Tables

**Table 2:** Table of baseline characteristics of included trials.

Study ID, Year	Clinical Trial Identifier	Number of Patients		Mean Age; Mean ± SD or Median (IQR)		Female Sex, *n* (%)		BMI; Mean ± SD or Median (IQR)		DM; *n* (%)		HTN; *n* (%)		Smoking; *n* (%)		LVEF; Mean (SD) or Median (IQR)		Follow-Up (Months)
		OCT	Angiography	OCT	Angiography	OCT	Angiography	OCT	Angiography	OCT	Angiography	OCT	Angiography	OCT	Angiography	OCT	Angiography	
ILUMIEN IV [12]	NCT03507777	992	981	65.6 ± 10.5	65.7 ± 10.4	200 (20.2)	231 (23.5)	-	-	360 (36.3)	342 (34.9)	707 (71.3)	734 (74.8)	198 (20)	192 (19.6)	55.1 (8.5)	55 (8.7)	2 years
OCTOBER [13]	NCT03171311	600	601	66.4 ± 10.5	66.2 ± 9.9	127 (21.2)	126 (21.0)	28.0 ± 4.6	28.2 ± 4.9	103 (17.2)	97 (16.1)	422 (70.3)	448 (74.5)	77 (12.8)	85 (14.1)	59.5 (50–60)	58.0 (50–60)	2 years
OCCUPI [14]	NCT03625908	803	801	64 (57−70)	64 (58−70)	157 (20%)	157 (20%)	24.8 (23.0−26.6)	24.6 (22.9−26.6)	261 (33%)	262 (33%)	466 (58%)	451 (56%)	149 (19%)	158 (20%)	59·5% (8·8)	59·7% (10·1)	1 year
Calipso Trial [15]	NCT05301218	65	69	72.0 (65.0−76.5)	74 (68.5−79.0)	13 (20)	12 (17)	26.8 (24.6–28.9)	26.0 (24.0–29.1)	24 (37)	27 (39)	46 (71)	43 (62)	8 (12)	10 (15)	60 (50–65)	60 (50–64)	1 month and 12 months
Renovate Complex PCI [16,17] *	NCT03381872	278	547	65.3	66	20.4	21.2	-	-	36.1	40.8	62.5	59	19.4	17.4	58.4	59.3	2.1 years

Abbreviations: BMI: body mass index; DM: diabetes mellitus; HTN: hypertension; LVEF: left ventricular ejection fraction; OCT: optical coherence tomography. * Represents baseline data from participants treated with IVUS-guided PCI in the source trial.

**Table 3:** Summary of pooled outcomes comparing optical coherence tomography (OCT)-guided percutaneous coronary intervention (PCI) versus angiography-guided PCI for complex coronary lesions. Effect estimates are reported as relative risks (RRs) for dichotomous outcomes and mean differences (MDs) for continuous outcomes, with corresponding 95% confidence intervals (CIs). Figures correspond to forest plots referenced in the manuscript. A *p*-value < 0.05 was considered statistically significant.

Outcome	Studies	Effect Estimate (95% CI)	*p* Value	Figure	Interpretation
Major Adverse Cardiovascular Events (MACEs)	5	RR = 0.71 (0.59–0.84)	0.0001	Figure 3a	OCT-guided PCI reduced MACE risk by 29%
Cardiac Mortality	4	RR = 0.43 (0.24–0.76)	0.003	Figure 3b	OCT-guided PCI lowered cardiac mortality by 57%
Target Lesion Revascularization (TLR)	3	RR = 0.53 (0.33–0.84)	0.007	Figure 3c	OCT-guided PCI decreased TLR by 47%
Stroke	2	RR = 0.17 (0.04–0.75)	0.02	Figure 4a	OCT-guided PCI reduced stroke risk by 83%
Stent Thrombosis	4	RR = 0.52 (0.31–0.86)	0.01	Figure 4b	OCT-guided PCI lowered stent thrombosis by 48%
All-Cause Mortality	4	RR = 0.58 (0.38–0.87)	0.009	Figure 4c	OCT-guided PCI reduced all-cause mortality by 42%
Procedural Duration	4	MD = 16.14 min (6.67–25.61)	0.0008	Figure S1	OCT-guided PCI prolonged procedure time by ~16 min
Ischemia-Driven TVR	4	RR = 0.67 (0.44–1.03)	0.07	Figure S2	No significant reduction (trend toward benefit)
Minimal Stent Area (MSA)	2	MD = 0.93 (–0.37–2.23)	0.16	Figure S3	No significant improvement in MSA
Myocardial Infarction (MI)	4	RR = 0.77 (0.57–1.03)	0.07	Figure S4	No significant reduction in MI

## Data Availability

Not applicable.

## Supplementary Materials

The following supporting information can be downloaded at: https://www.mdpi.com/article/10.3390/diagnostics15151907/s1, Figure S1: Forest plot for total procedural duration, Figure S2: Forest plot for ischemia driven TVR, Figure S3: Forest plot for minimal stent area, Figure S4: Forest plot for myocardial infarction; Table S1: PRISMA 2020 Checklist, Table S2: Search strategy for electronic databases, Table S3: Risk of Bias assessment across studies.

## Abbreviations

ACS	Acute coronary syndrome
BMI	Body mass index
CTO	Chronic total occlusion
DM	Diabetes mellitus
HTN	Hypertension
LVEF	Left ventricular ejection fraction.
MACEs	Major adverse cardiovascular events
MI	Myocardial infarction
OCT	Optical coherence tomography
PCI	Percutaneous coronary intervention
RCTs	Randomized controlled trials
RR	Risk ratio
STEMI	ST-elevation myocardial infarction
TLR	Target lesion revascularization
CKD	Chronic kidney disease
CA-AKI	Contrast-associated acute kidney injury
