# Supplementary material for: Optical Coherence Tomography-Guided vs. Angiography-Guided Percutaneous Coronary Intervention for Complex Coronary Lesions: A Systematic Review and Meta-Analysis
Source: Diagnostics (Basel). 2025 Jul 30;15(15):1907. doi: 10.3390/diagnostics15151907 (PMC12345747; doi:10.3390/diagnostics15151907)

SUPPLEMENTARY MATERIAL

Optical Coherence Tomography-Guided vs. Angiography-Guided Percutaneous Coronary Intervention for Complex Coronary Lesions: A Systematic Review and Meta-Analysis.

Muhammad Hamza Shuja, MBBS; Muhammad Ahmed, MBBS; Ramish Hannat; MBBS; Laiba Khurram, MBBS; Hamza Ali Hasnain Sheikh, MBBS; Syed Hasan Shuja, MBBS; Adarsh Raja, MBBS; Jawad Ahmed, MD; Kriti Soni, MD; Shariq Ahmad Wani, MD; Aman Goyal, MD; Bala Pushparaji, MD; Ali Hassan, MBBS; Raheel Ahmed, MBBS, MRCP, PhD; Hritvik Jain, MBBS

Supplementary Table S1. PRISMA 2020 Checklist

Section and Topic	Item #	Checklist item	Location where item is reported
**TITLE**			
Title	1	Identify the report as a systematic review.	1
**ABSTRACT**			
Abstract	2	See the PRISMA 2020 for Abstracts checklist.	5
**INTRODUCTION**			
Rationale	3	Describe the rationale for the review in the context of existing knowledge.	7-8
Objectives	4	Provide an explicit statement of the objective(s) or question(s) the review addresses.	8
**METHODS**			
Eligibility criteria	5	Specify the inclusion and exclusion criteria for the review and how studies were grouped for the syntheses.	9
Information sources	6	Specify all databases, registers, websites, organisations, reference lists and other sources searched or consulted to identify studies. Specify the date when each source was last searched or consulted.	9
Search strategy	7	Present the full search strategies for all databases, registers, and websites, including any filters and limits used.	9
Selection process	8	Specify the methods used to decide whether a study met the inclusion criteria of the review, including how many reviewers screened each record and each report retrieved, whether they worked independently, and if applicable, details of automation tools used in the process.	9
Data collection process	9	Specify the methods used to collect data from reports, including how many reviewers collected data from each report, whether they worked independently, any processes for obtaining or confirming data from study investigators, and if applicable, details of automation tools used in the process.	10
Data items	10a	List and define all outcomes for which data were sought. Specify whether all results that were compatible with each outcome domain in each study were sought (e.g. for all measures, time points,	10

Section and Topic	Item #	Checklist item	Location where item is reported
		analyses), and if not, the methods used to decide which results to collect.	
	10b	List and define all other variables for which data were sought (e.g. participant and intervention characteristics, funding sources). Describe any assumptions made about any missing or unclear information.	10
Study risk of bias assessment	11	Specify the methods used to assess risk of bias in the included studies, including details of the tool(s) used, how many reviewers assessed each study and whether they worked independently, and if applicable, details of automation tools used in the process.	10
Effect measures	12	Specify for each outcome the effect measure(s) (e.g. risk ratio, mean difference) used in the synthesis or presentation of results.	11
Synthesis methods	13a	Describe the processes used to decide which studies were eligible for each synthesis (e.g. tabulating the study intervention characteristics and comparing against the planned groups for each synthesis (item #5)).	NA
	13b	Describe any methods required to prepare the data for presentation or synthesis, such as handling of missing summary statistics, or data conversions.	NA
	13c	Describe any methods used to tabulate or visually display results of individual studies and syntheses.	11
	13d	Describe any methods used to synthesize results and provide a rationale for the choice(s). If meta-analysis was performed, describe the model(s), method(s) to identify the presence and extent of statistical heterogeneity, and software package(s) used.	11
	13e	Describe any methods used to explore possible causes of heterogeneity among study results (e.g. subgroup analysis, meta-regression).	11
	13f	Describe any sensitivity analyses conducted to assess robustness of the synthesized results.	11

Section and Topic	Item #	Checklist item	Location where item is reported
Reporting bias assessment	14	Describe any methods used to assess risk of bias due to missing results in a synthesis (arising from reporting biases).	NA
Certainty assessment	15	Describe any methods used to assess certainty (or confidence) in the body of evidence for an outcome.	NA
**RESULTS**			
Study selection	16a	Describe the results of the search and selection process, from the number of records identified in the search to the number of studies included in the review, ideally using a flow diagram.	12
	16b	Cite studies that might appear to meet the inclusion criteria, but which were excluded, and explain why they were excluded.	12
Study characteristics	17	Cite each included study and present its characteristics.	12
Risk of bias in studies	18	Present assessments of risk of bias for each included study.	12
Results of individual studies	19	For all outcomes, present, for each study: (a) summary statistics for each group (where appropriate) and (b) an effect estimate and its precision (e.g. confidence/credible interval), ideally using structured tables or plots.	12-13
Results of syntheses	20a	For each synthesis, briefly summarise the characteristics and risk of bias among contributing studies.	12-13
	20b	Present results of all statistical syntheses conducted. If meta-analysis was done, present for each the summary estimate and its precision (e.g. confidence/credible interval) and measures of statistical heterogeneity. If comparing groups, describe the direction of the effect.	12-13
	20c	Present results of all investigations of possible causes of heterogeneity among study results.	12-13
	20d	Present results of all sensitivity analyses conducted to assess the robustness of the synthesized results.	12-13
Reporting biases	21	Present assessments of risk of bias due to missing results (arising from reporting biases) for each synthesis assessed.	NA

Section and Topic	Item #	Checklist item	Location where item is reported
Certainty of evidence	22	Present assessments of certainty (or confidence) in the body of evidence for each outcome assessed.	NA
**DISCUSSION**			
Discussion	23a	Provide a general interpretation of the results in the context of other evidence.	14-15
	23b	Discuss any limitations of the evidence included in the review.	17-18
	23c	Discuss any limitations of the review processes used.	17
	23d	Discuss implications of the results for practice, policy, and future research.	15-18
**OTHER INFORMATION**			
Registration and protocol	24a	Provide registration information for the review, including register name and registration number, or state that the review was not registered.	PROSPERO CRD42024599058
	24b	Indicate where the review protocol can be accessed, or state that a protocol was not prepared.	PROSPERO CRD42024599058
	24c	Describe and explain any amendments to information provided at registration or in the protocol.	NA
Support	25	Describe sources of financial or non-financial support for the review, and the role of the funders or sponsors in the review.	Title Page
Competing interests	26	Declare any competing interests of review authors.	Title Page
Availability of data, code, and other materials	27	Report which of the following are publicly available and where they can be found: template data collection forms; data extracted from included studies; data used for all analyses; analytic code; any other materials used in the review.	Title Page

Supplementary Table S2. Search strategy for electronic databases

PubMed (n=1135)	("OCT"[All Fields] OR "optical coherence tomography"[All Fields] OR "OCT-guided PCI"[All Fields] OR "optical coherence tomography-guided PCI"[All Fields]) AND ("angiography"[All Fields] OR "angiography guided pci"[All Fields] OR "angiography guided pci"[All Fields]) AND ("complex coronary lesions"[All Fields] OR "complex lesions"[All Fields] OR "left main disease"[All Fields] OR "left main coronary artery disease"[All Fields] OR "bifurcation lesions"[All Fields] OR "chronic total occlusion"[All Fields] OR "CTO"[All Fields] OR "long lesions"[All Fields] OR "calcified lesions"[All Fields] OR "severely calcified lesions"[All Fields] OR "multivessel disease"[All Fields] OR "tandem lesions"[All Fields] OR "ostial lesions"[All Fields] OR "small vessel disease"[All Fields] OR "thrombotic lesions"[All Fields])
Cochrane Library (n=23)	(optical coherence tomography) AND (angiography-guided OR conventional) AND (complex coronary lesions OR complex lesions)
SCOPUS (N=919)	((TITLE-ABS-KEY (OCT) OR TITLE-ABS-KEY ("optical coherence tomography") OR TITLE-ABS-KEY ("OCT guided PCI") OR TITLE-ABS-KEY ("optical coherence tomography guided PCI")) AND (TITLE-ABS-KEY (angiography) OR TITLE-ABS-KEY ("angiography guided PCI")) AND (TITLE-ABS-KEY ("complex coronary lesions") OR TITLE-ABS-KEY ("complex lesions") OR TITLE-ABS-KEY ("left main disease") OR TITLE-ABS-KEY ("left main coronary artery disease") OR TITLE-ABS-KEY ("bifurcation lesions") OR TITLE-ABS-KEY ("chronic total occlusion") OR TITLE-ABS-KEY (CTO) OR TITLE-ABS-KEY ("long lesions") OR TITLE-ABS-KEY ("calcified lesions") OR TITLE-ABS-KEY ("severely calcified lesions") OR TITLE-ABS-KEY ("multivessel disease") OR TITLE-ABS-KEY ("tandem lesions") OR TITLE-ABS-KEY ("ostial lesions") OR TITLE-ABS-KEY ("small vessel disease") OR TITLE-ABS-KEY ("thrombotic lesions"))
Clinicaltrials.gov (n=6)	optical coherence tomography) AND (angiography-guided) AND (complex coronary lesion)

Supplementary Table S3: Risk of Bias assessment across studies

Bias Domain	ILLUMENIV. (2023)	OCTOBER. (2023)	OCCUPI. (2024)	CALIPSO (2025)	RENOVATE COMPLEX PCI (2024)
**Random sequence generation** (selection bias)	**Low Risk** – Random 1:1 assignment with block sizes.	**Low Risk** – Web-based randomization with permuted blocks.	**Low Risk** – Computer-generated via web system.	**Low Risk** – Secure online randomization post-angiography.	**Low Risk** – Online randomization with stratified block sizes.
**Allocation concealment** (selection bias)	**Unclear Risk** – No specific method stated.	**Low Risk** – Concealed external web system.	**Low Risk** – Web-based system by external programmer.	**Low Risk** – Secure web-based system.	**Low Risk** – Web-based permuted blocks post-angiography.
**Blinding of participants and personnel** (performance bias)	**Low Risk** – Single-blinded design.	**High Risk** – Open-label trial.	**High Risk** – Open-label; full awareness.	**High Risk** – Open-label; known allocation.	**High Risk** – Prospective open-label multicenter trial.
**Blinding of outcome assessment** (detection bias)	**Low Risk** – Independent clinical group.	**Low Risk** – Independent clinical committee.	**Low Risk** – Independent blinded panel.	**Low Risk** – Core lab analysis of OCT data.	**Low Risk** – Blinded adjudication committee and core labs.
**Incomplete outcome data** (attrition bias)	**Low Risk** – ITT analysis, all participants accounted for.	**Low Risk** – Minimal follow-up loss.	**Low Risk** – ITT analysis with minimal loss.	**Low Risk** – 93.7% follow-up; pre-specified exclusion criteria.	**Low Risk** – Complete follow-up; protocol violations included in ITT.
**Selective reporting** (reporting bias)	**Low Risk** – Pre-specified outcomes reported.	**Low Risk** – All registered outcomes reported.	**Low Risk** – Outcomes aligned with trial protocol.	**Low Risk** – All outcomes reported (NCT05301218).	**Low Risk** – Registered and published protocol

					(NCT03381872).
**Other bias**	**Low Risk** – Rigorous methodology, no evidence of other bias.	**Low Risk** – No additional biases noted.	**Low Risk** – No funding or COI issues.	**Low Risk** – Ethical conduct and balanced baseline characteristics.	**Low Risk** – Ethical approval and transparent funding; no bias

Supplementary Figure 1a: Forest plot for total procedural duration

Study or Subgroup	OCT-Guided Mean	OCT-Guided SD	OCT-Guided Total	Angiography-Guided Mean	Angiography-Guided SD	Angiography-Guided Total	Weight	Mean Difference IV, Random, 95% CI
CALIPSO	67	27.29	65	64	27.26	69	21.6%	3.00 [-6.24, 12.24]
ILUMEN IV	72	39.2	992	53.9	37.1	981	26.2%	18.10 [14.73, 21.47]
OCCUPI	54.33	22.28	778	43.67	20.8	762	26.8%	10.66 [8.51, 12.81]
OCTOBER	114.33	44.59	600	83.33	37.16	601	25.5%	31.00 [26.36, 35.64]
**Total (95% CI)**			**2435**			**2413**	**100.0%**	**16.14 [6.67, 25.61]**

Heterogeneity: Tau² = 85.98; Chi² = 70.94, df = 3 (P < 0.00001); I² = 96%
Test for overall effect: Z = 3.34 (P = 0.0008)

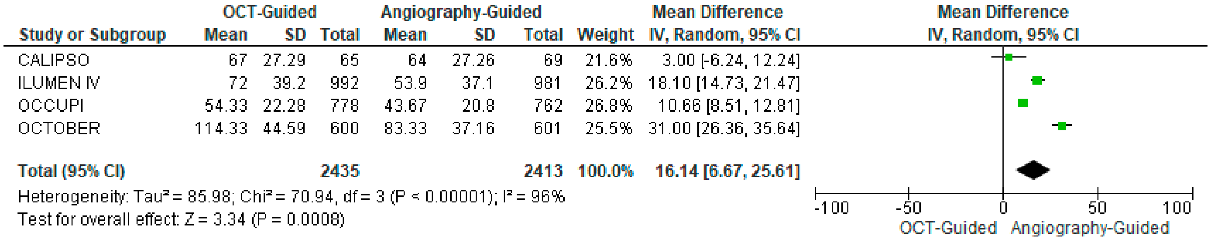

Supplementary Figure S1b: Forest plot from sensitivity analysis for total procedural duration.

Study or Subgroup	OCT-Guided Mean	OCT-Guided SD	OCT-Guided Total	Angiography-Guided Mean	Angiography-Guided SD	Angiography-Guided Total	Weight	Mean Difference IV, Random, 95% CI
CALIPSO	67	27.29	65	64	27.26	69	23.1%	3.00 [-6.24, 12.24]
ILUMEN IV	72	39.2	992	53.9	37.1	981	37.4%	18.10 [14.73, 21.47]
OCCUPI	54.33	22.28	778	43.67	20.8	762	39.6%	10.66 [8.51, 12.81]
OCTOBER	114.33	44.59	600	83.33	37.16	601	0.0%	31.00 [26.36, 35.64]
**Total (95% CI)**			**1835**			**1812**	**100.0%**	**11.67 [4.99, 18.36]**

Heterogeneity: Tau² = 28.17; Chi² = 17.49, df = 2 (P = 0.0002); I² = 89%
Test for overall effect: Z = 3.42 (P = 0.0006)

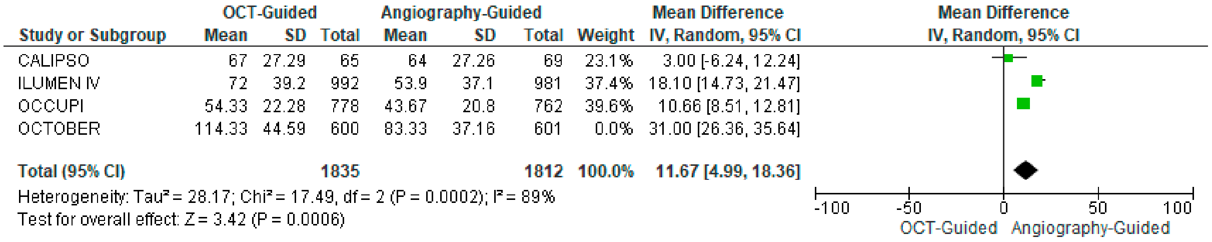

Supplementary Figure S2a: Forest plot for ischemia driven TVR

Study or Subgroup	OCT-Guided Events	OCT-Guided Total	Angiography-Guided Events	Angiography-Guided Total	Weight	Risk Ratio M-H, Random, 95% CI
ILUMEN IV	53	992	55	981	34.2%	0.95 [0.66, 1.38]
OCCUPI	12	803	33	801	22.0%	0.36 [0.19, 0.70]
OCTOBER	17	600	27	601	24.1%	0.63 [0.35, 1.14]
RENOVATE-COMPLEX-PCI	10	278	25	547	19.8%	0.79 [0.38, 1.62]
**Total (95% CI)**		**2673**		**2930**	**100.0%**	**0.67 [0.44, 1.03]**
Total events	92		140			

Heterogeneity: Tau² = 0.10; Chi² = 6.73, df = 3 (P = 0.08); I² = 55%
Test for overall effect: Z = 1.84 (P = 0.07)

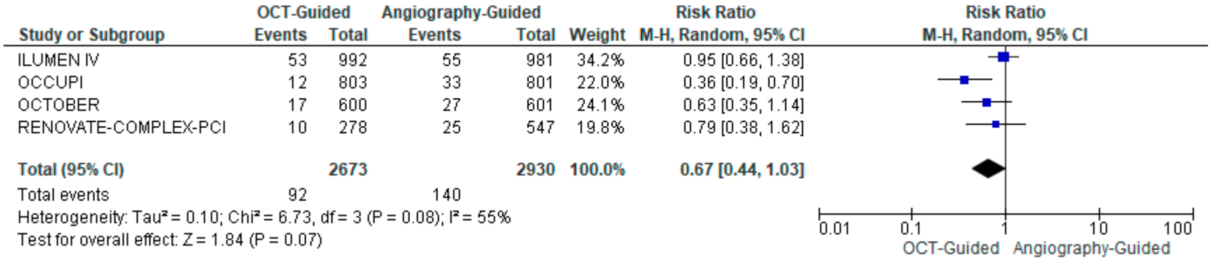

Supplementary Figure S2b: Forest Plot from sensitivity analysis for ischemia driven TVR.

Study or Subgroup	OCT-Guided Events	OCT-Guided Total	Angiography-Guided Events	Angiography-Guided Total	Weight	Risk Ratio M-H, Random, 95% CI
ILUMEN IV	53	992	55	981	61.0%	0.95 [0.66, 1.38]
OCCUPI	12	803	33	801	0.0%	0.36 [0.19, 0.70]
OCTOBER	17	600	27	601	23.1%	0.63 [0.35, 1.14]
RENOVATE-COMPLEX-PCI	10	278	25	547	15.9%	0.79 [0.38, 1.62]
**Total (95% CI)**		**1870**		**2129**	**100.0%**	**0.84 [0.63, 1.12]**
Total events	80		107			

Heterogeneity: Tau² = 0.00; Chi² = 1.37, df = 2 (P = 0.50); I² = 0%
Test for overall effect: Z = 1.19 (P = 0.23)

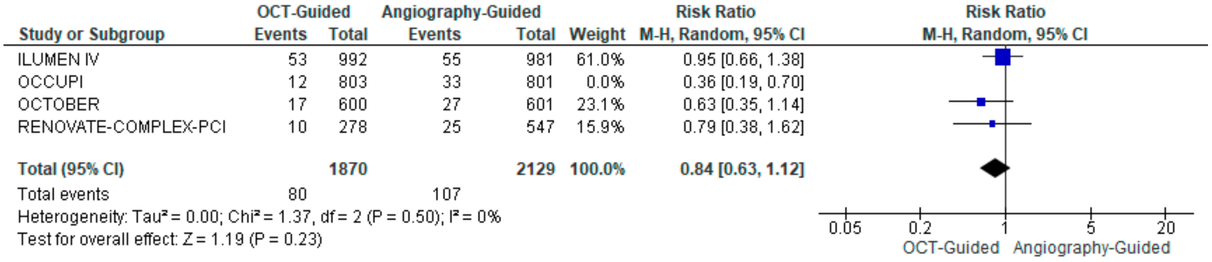

Supplementary Figure S3: Forest plot for minimal stent area

Study or Subgroup	OCT-Guided Mean	OCT-Guided SD	OCT-Guided Total	Angiography-Guided Mean	Angiography-Guided SD	Angiography-Guided Total	Weight	Mean Difference IV, Random, 95% CI
CALIPSO	6.7	1.97	65	5.07	1.51	69	47.6%	1.63 [1.03, 2.23]
ILUMEN IV	5.56	1.95	994	5.26	1.81	1057	52.4%	0.30 [0.14, 0.46]
**Total (95% CI)**			**1059**			**1126**	**100.0%**	**0.93 [-0.37, 2.23]**

Heterogeneity: $Tau^2 = 0.83$; $Chi^2 = 17.75$, df = 1 ($P < 0.0001$); $I^2 = 94\%$
Test for overall effect: Z = 1.40 (P = 0.16)

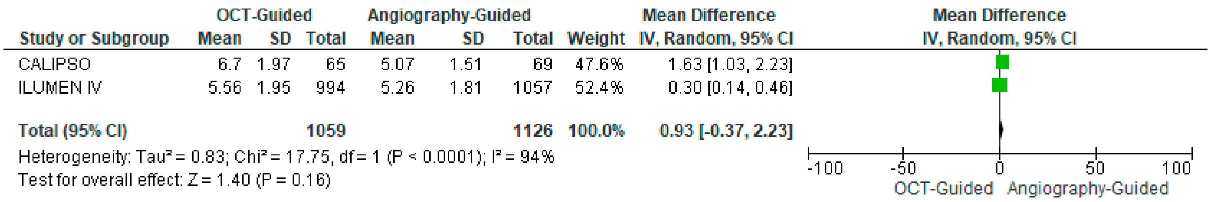

Supplementary Figure S4: Forest plot for myocardial infarction

Study or Subgroup	OCT-Guided Events	OCT-Guided Total	Angiography-Guided Events	Angiography-Guided Total	Weight	Risk Ratio M-H, Random, 95% CI
ILUMEN IV	25	992	35	981	24.1%	0.71 [0.43, 1.17]
OCCUPI	29	803	40	801	26.9%	0.72 [0.45, 1.15]
OCTOBER	54	600	54	601	37.5%	1.00 [0.70, 1.44]
RENOVATE-COMPLEX-PCI	7	278	32	547	11.5%	0.43 [0.19, 0.96]
**Total (95% CI)**		**2673**		**2930**	**100.0%**	**0.77 [0.57, 1.03]**
Total events	115		161			

Heterogeneity: $Tau^2 = 0.03$; $Chi^2 = 4.21$, df = 3 (P = 0.24); $I^2 = 29\%$
Test for overall effect: Z = 1.79 (P = 0.07)

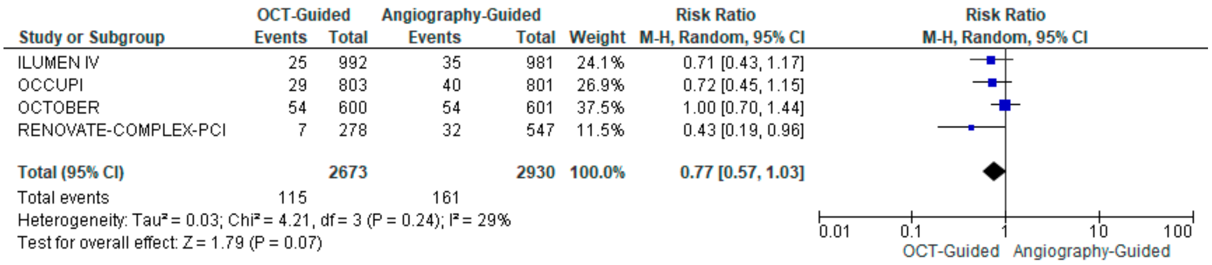

Supplement: Supplementary file 1 [file diagnostics-15-01907-s001.zip › diagnostics-3765606-supplementary.pdf]
